# Emerging infectious diseases associated with bat viruses

**DOI:** 10.1007/s11427-013-4517-x

**Published:** 2013-08-07

**Authors:** ZhengLi Shi

**Affiliations:** Center for Emerging Infectious Diseases, State Key Laboratory of Virology, Wuhan Institute of Virology, Chinese Academy of Sciences, Wuhan, 430071 China

**Keywords:** emerging infectious disease, bat virus, natural reservoirs

## Abstract

Bats play important roles as pollen disseminators and pest predators. However, recent interest has focused on their role as natural reservoirs of pathogens associated with emerging infectious diseases. Prior to the outbreak of severe acute respiratory syndrome (SARS), about 60 bat virus species had been reported. The number of identified bat viruses has dramatically increased since the initial SARS outbreak, and most are putative novel virus species or genotypes. Serious infectious diseases caused by previously identified bat viruses continue to emerge throughout in Asia, Australia, Africa and America. Intriguingly, bats infected by these different viruses seldom display clinical symptoms of illness. The pathogenesis and potential threat of bat-borne viruses to public health remains largely unknown. This review provides a brief overview of bat viruses associated with emerging human infectious diseases.

## References

[1] Woo P C Y, Lau S K P, Lam C S F (2012). Discovery of seven novel mammalian and avian coronaviruses in the genus *Deltacoronavirus* supports bat coronaviruses as the gene source of Alphacoronavirus and Betacoronavirus and avian coronaviruses as the gene source of Gammacoronavirus and Deltacoronavirus. J Virol.

[2] de Groot R, Baker S, Baric R (2012). Family Coronaviridae. Virus Taxonomy: Ninth Report of the International Committee on Taxonomy of Viruses.

[3] Ksiazek T G, Erdman D, Goldsmith C S (2003). A novel coronavirus associated with severe acute respiratory syndrome. N Engl J Med.

[4] Drosten C, Gunther S, Preiser W (2003). Identification of a novel coronavirus in patients with severe acute respiratory syndrome. N Engl J Med.

[5] Guan Y, Zheng B J, He Y Q (2003). Isolation and characterization of viruses related to the SARS coronavirus from animals in southern China. Science.

[6] Wang M, Yan M, Xu H (2005). SARS-CoV infection in a restaurant from palm civet. Emerg Infect Dis.

[7] Tu C, Crameri G, Kong X (2004). Antibodies to SARS coronavirus in civets. Emerg Infect Dis.

[8] Kan B, Wang M, Jing H (2005). Molecular evolution analysis and geographic investigation of severe acute respiratory syndrome coronavirus-like virus in palm civets at an animal market and on farms. J Virol.

[9] Poon L L M, Chu D K W, Chan K H (2005). Identification of a novel coronavirus in bats. J Virol.

[10] Li W D, Shi Z L, Yu M (2005). Bats are natural reservoirs of SARS-like coronaviruses. Science.

[11] Lau S K P, Woo P C Y, Li K S M (2005). Severe acute respiratory syndrome coronavirus-like virus in Chinese horseshoe bats. Proc Natl Acad Sci USA.

[12] Drexler J F, Gloza-Rausch F, Glende J (2010). Genomic characterization of severe acute respiratory syndrome-related coronavirus in European bats and classification of coronaviruses based on partial RNA-dependent RNA polymerase gene sequences. J Virol.

[13] Tong S, Conrardy C, Ruone S (2009). Detection of novel SARS-like and other coronaviruses in bats from Kenya. Emerg Infect Dis.

[14] Yuan J, Hon C C, Li Y (2010). Intraspecies diversity of SARS-like coronaviruses in Rhinolophus sinicus and its implications for the origin of SARS coronaviruses in humans. J Gen Virol.

[15] Ren W, Li W D, Yu M (2006). Full-length genome sequences of two SARS-like coronaviruses in horseshoe bats and genetic variation analysis. J Gen Virol.

[16] Quan P L, Firth C, Street C (2010). Identification of a severe acute respiratory syndrome coronavirus-like virus in a leaf-nosed bat in Nigeria. MBio.

[17] Ren W, Qu X X, Li W D (2008). Difference in receptor usage between severe acute respiratory syndrome (SARS) coronavirus and SARSlike coronavirus of bat origin. J Virol.

[18] Hon C C, Lam T Y, Shi Z L (2008). Evidence of the recombinant origin of a bat severe acute respiratory syndrome (SARS)-like coronavirus and its implications on the direct ancestor of SARS coronavirus. J Virol.

[19] WHO. Coronavirus infections. http://www.who.int/csr/disease/coronavirus_infections/en/

[20] Zaki A M, van Boheemen S, Bestebroer T M (2012). Isolation of a novel coronavirus from a man with pneumonia in Saudi Arabia. N Engl J Med.

[21] Lau S K, Li K S, Tsang A K (2013). Genetic characterization of *Betacoronavirus* lineage C viruses in bats revealed marked sequence divergence in the spike protein of *Pipistrellus* bat coronavirus HKU5 in Japanese pipistrelle: implications on the origin of the novel Middle East Respiratory Syndrome Coronavirus. J Virol.

[22] van Boheemen S, de Graaf M, Lauber C (2012). Genomic characterization of a newly discovered coronavirus associated with acute respiratory distress syndrome in humans. MBio.

[23] Raj V S, Mou H, Smits S L (2013). Dipeptidyl peptidase 4 is a functional receptor for the emerging human coronavirus-EMC. Nature.

[24] Lu GW, Liu D (2012). SARS-like virus in the middle east: A truly bat-related coronavirus causing human diseases. Protein Cell.

[25] van der Hoek L, Pyrc K, Jebbink M F (2004). Identification of a new human coronavirus. Nat Med.

[26] Cavallar J J, Monto A S (1970). Community-wide outbreak of infection with a 229E-like coronavirus in Tecumseh, Michigan. J Infect Dis.

[27] Pfefferle S, Oppong S, Drexler J F (2009). Distant relatives of severe acute respiratory syndrome coronavirus and close relatives of human coronavirus 229E in bats, Ghana. Emerg Infect Dis.

[28] Huynh J, Li S, Yount B (2012). Evidence supporting a zoonotic origin of human coronavirus strain NL63. J Virol.

[29] Johnson N, Vos A, Freuling C (2010). Human rabies due to lyssavirus infection of bat origin. Vet Microbiol.

[30] Rupprecht C E, Turmelle A, Kuzmin I V (2011). A perspective on lyssavirus emergence and perpetuation. Cur Opin Virol.

[31] Tang X, Luo M, Zhang S (2005). Pivotal role of dogs in rabies transmission, China. Emerg Infect Dis.

[32] Murray K, Selleck P, Hooper P (1995). A morbillivirus that caused fatal disease in horses and humans. Science.

[33] Field H, Crameri G, Kung N Y (2012). Ecological aspects of Hendra virus. Cur Top Microbiol Immunol.

[34] Chua K B, Bellini W J, Rota P A (2000). Nipah virus: a recently emergent deadly paramyxovirus. Science.

[35] Lo M K, Rota P A (2008). The emergence of Nipah virus, a highly pathogenic paramyxovirus. J Clin Virol.

[36] Luby P S, Hossain M J, Gurley E S (2009). Recurrent zoonotic transmission of Nipah virus into humans, Bangladesh, 2001–2007. Emerg Infect Dis.

[37] Tan C T, Goh K J, Wong K T (2002). Relapsed and late-onset Nipah encephalitis. Ann Neurol.

[38] Wang L F, Yu M, Hansson E (2000). The exceptionally large genome of Hendra virus: support for creation of a new genus within the family Paramyxoviridae. J Virol.

[39] Reynes J M, Counor D, Ong S (2005). Nipah virus in lyle’s flying foxes, Cambodia. Emerg Infect Dis.

[40] Wacharapluesadee S, Lumlertdacha B, Boongird K (2005). Bat Nipah virus, Thailand. Emerg Infect Dis.

[41] Drexler J F, Corman V M, Muller M A (2012). Bats host major mammalian paramyxoviruses. Nat Commun.

[42] Shi Z (2010). Bat and virus. Protein Cell.

[43] Calisher C H, Childs J E, Field H E (2006). Bats: important reservoir hosts of emerging viruses. Clin Microbiol Rev.

[44] Wong S, Lau S, Woo P (2007). Bats as a continuing source of emerging infections in humans. Rev Med Virol.

[45] Barrette R W, Xu L, Rowland J M (2011). Current perspectives on the phylogeny of Filoviridae. Infect Genet Evol.

[46] Slenczka W, Klenk H D (2007). Forty years of Marburg virus. J Infect Dis.

[47] Towner J S, Amman B R, Sealy T K (2009). Isolation of genetically diverse Marburg viruses from Egyptian fruit bats. PLoS Pathog.

[48] Leroy E M, Kumulungui B, Pourrut X (2005). Fruit bats as reservoirs of Ebola virus. Nature.

[49] Towner J S, Pourrut X, Albarino C G (2007). Marburg virus infection detected in a common African bat. PLoS ONE.

[50] Hayman D T, Emmerich P, Yu M (2010). Long-term survival of an urban fruit bat seropositive for Ebola and Lagos bat viruses. PLoS ONE.

[51] Pourrut X, Souris M, Towner J S (2009). Large serological survey showing cocirculation of ebola and marburg viruses in gabonese bat populations, and a high seroprevalence of both viruses in Rousettus aegyptiacus. BMC Infect Dis.

[52] Biek R, Walsh P D, Leroy E M (2006). Recent common ancestry of Ebola zaire virus found in a bat reservoir. PLoS Pathog.

[53] Gard G P, Marshall I D (1973). Nelson bay virus. A novel reovirus. Arch Gesamte Virusforsch.

[54] Pritchard L I, Chua K B, Cummins D (2006). Pulau virus: a new member of the Nelson bay orthoreovirus species isolated from fruit bats in Malaysia. Arch Virol.

[55] Du L, Lu Z, Fan Y (2010). Xi river virus, a new bat reovirus isolated in southern China. Arch Virol.

[56] Chua K B, Voon K, Crameri G (2008). Identification and characterization of a new orthoreovirus from patients with acute respiratory infections. PLoS ONE.

[57] Chua K B, Crameri G, Hyatt A (2007). A previously unknown reovirus of bat origin is associated with an acute respiratory disease in humans. Proc Nat Acad Sci USA.

[58] Wong A H, Cheng P K, Lai M Y (2012). Virulence potential of fusogenic orthoreoviruses. Emerg Infect Dis.

[59] Kohl C, Lesnik R, Brinkmann A (2012). Isolation and characterization of three mammalian orthoreoviruses from European bats. PLoS ONE.

[60] Lelli D, Moreno A, Lavazza A (2012). Identification of mammalian orthoreovirus type 3 in Italian bats. Zoo Pub Health.

[61] Li Y, Ge X, Zhang H (2010). Host range, prevalence, and genetic diversity of adenoviruses in bats. J Virol.

[62] Maeda K, Hondo E, Terakawa J (2008). Isolation of novel adenovirus from fruit bat (*Pteropus dasymallus yayeyamae*). Emerg Infect Dis.

[63] Xiao J, Li J, Hu G (2011). Isolation and phylogenetic characterization of bat astroviruses in southern China. Arch Virol.

[64] Zhu H C, Chu D K, Liu W (2009). Detection of diverse astroviruses from bats in china. J Gen Virol.

[65] Tsuda S, Watanabe S, Masangkay J S (2012). Genomic and serological detection of bat coronavirus from bats in the Philippines. Arch Virol.

[66] Lau S K P, Li K S M, Tsang A K L (2012). Recent transmission of a novel alphacoronavirus, bat coronavirus HKU10, from leschenault’s rousettes to pomona leaf-nosed bats: first evidence of interspecies transmission of coronavirus between bats of different suborders. J Virol.

[67] August T A, Mathews F, Nunn M A (2012). Alphacoronavirus detected in bats in the United Kingdom. Vector-Borne Zoonot.

[68] Tao Y, Tang K, Shi M (2012). Genomic characterization of seven distinct bat coronaviruses in Kenya. Virus Res.

[69] Lau S K P, Poon R W S, Wong B H L (2010). Coexistence of different genotypes in the same bat and serological characterization of rousettus bat coronavirus HKU9 belonging to a novel betacoronavirus subgroup. J Virol.

[70] Watanabe S, Masangkay J S, Nagata N (2010). Bat coronaviruses and experimental infection of bats, the Philippines. Emerg Infect Dis.

[71] Donaldson E F, Haskew A N, Gates J E (2010). Metagenomic analysis of the viromes of three north American bat species: viral diversity among different bat species that share a common habitat. J Virol.

[72] Ge X, Li J, Peng C (2012). Genetic diversity of novel circular ssDNA viruses in bats in China. J Gen Virol.

[73] Negredo A, Palacios G, Vazquez-Moron S (2011). Discovery of an Ebolavirus-like filovirus in Europe. PLoS Pathog.

[74] Wu Z, Ren X, Yang L (2012). Virome analysis for identification of novel mammalian viruses in bat species from Chinese provinces. J Virol.

[75] Quan P L, Firth C, Conte J M (2013). Bats are a major natural reservoir for hepaciviruses and pegiviruses. Proc Natil Acad Sci USA.

[76] He B, Li Z, Yang F (2013). Virome profiling of bats from Myanmar by metagenomic analysis of tissue samples reveals more novel mammalian viruses. PLoS ONE.

[77] Drexler J F, Seelen A, Corman V M (2012). Bats worldwide carry hepatitis E virus-related viruses that form a putative novel genus within the family Hepeviridae. J Virol.

[78] Zhang H, Todd S, Tachedjian M (2012). A novel bat herpesvirus encodes homologues of major histocompatibility complex classes I and II, c-type lectin, and a unique family of immune-related genes. J Virol.

[79] Tong S, Li Y, Rivailler P (2012). A distinct lineage of influenza a virus from bats. Proc Natil Acad Sci USA.

[80] Sun X, Shi Y, Lu X (2013). Bat-derived influenza hemagglutinin H17 does not bind canonical avian or human receptors and most likely uses a unique entry mechanism. Cell Rep.

[81] Ge X, Li Y, Yang X (2012). Metagenomic analysis of viruses from bat fecal samples reveals many novel viruses in insectivorous bats in China. J Virol.

[82] Lau S K, Woo P C, Lai K K (2011). Complete genome analysis of three novel picornaviruses from diverse bat species. J Virol.

[83] McKnight C A, Wise A G, Maes R K (2006). Papillomavirusassociated basosquamous carcinoma in an Egyptian fruit bat (*Rousettus aegyptiacus*). J Zoo Wildl Med.

[84] Li L, Victoria J G, Wang C (2010). Bat guano virome: predominance of dietary viruses from insects and plants plus novel mammalian viruses. J Virol.

[85] Zhang G, Cowled C, Shi Z (2013). Comparative analysis of bat genomes provides insight into the evolution of flight and immunity. Science.

[86] Turnell A S, Grand R J (2012). DNA viruses and the cellular DNA-damage response. J Gen Virol.

